# *N*-Heterocyclic Carbene Iron Complexes as Anticancer Agents: In Vitro and In Vivo Biological Studies

**DOI:** 10.3390/molecules26185535

**Published:** 2021-09-12

**Authors:** Oscar A. Lenis-Rojas, Sandra Cordeiro, Marta Horta-Meireles, Jhonathan Angel Araujo Fernández, Sabela Fernández Vila, Juan Andrés Rubiolo, Pablo Cabezas-Sainz, Laura Sanchez, Alexandra R. Fernandes, Beatriz Royo

**Affiliations:** 1 ITQB NOVA, Instituto de Tecnologia Química e Biológica António Xavier, Av. da República, 2780-157 Oeiras, Portugal; m.meireles@itqb.unl.pt; 2 UCIBIO, Departamento Ciências da Vida, NOVA School of Science and Technology, NOVA University, Campus de Caparica, 2829-516 Caparica, Portugal; si.cordeiro@campus.fct.unl.pt; 3 Associate Laboratory i4HB—Institute for Health and Bioeconomy, NOVA School of Science and Technology, NOVA University, 2819-516 Caparica, Portugal; 4 Departamento de Zoología Genética y Antropología Física, Facultad de Veterinaria, Universidad de Santiago de Compostela, Campus de Lugo, 27002 Lugo, Spain; araujof27@gmail.com (J.A.A.F.); sabela.fernandez.vila@rai.usc.es (S.F.V.); ja.rubiolo@usc.es (J.A.R.); pablo.cabezas@usc.es (P.C.-S.); lauraelena.sanchez@usc.es (L.S.); 5 Laboratory of Zebrafish, Department of Medical Genetics and Genomic Medicine—School of Medical Sciences, University of Campinas (UNICAMP), Campinas 13083-970, SP, Brazil; 6 Facultad de Ciencias Bioquímicas y Farmacéuticas-Centro Científico y Tecnológico Acuario del Río Paraná, Universidad Nacional de Rosario, Rosario 2000, Argentina; 7 Preclinical Animal Models Group, Health Research Institute of Santiago de Compostela (IDIS), 5706 Santiago de Compostela, Spain

**Keywords:** *N*-heterocyclic carbene, iron(II)–NHC complexes, anticancer activity, zebrafish

## Abstract

Cisplatin and its derivatives are commonly used in chemotherapeutic treatments of cancer, even though they suffer from many toxic side effects. The problems that emerge from the use of these metal compounds led to the search for new complexes capable to overcome the toxic side effects. Here, we report the evaluation of the antiproliferative activity of Fe(II) cyclopentadienyl complexes bearing *n*-heterocyclic carbene ligands in tumour cells and their in vivo toxicological profile. The in vitro antiproliferative assays demonstrated that complex **Fe1** displays the highest cytotoxic activity both in human colorectal carcinoma cells (HCT116) and ovarian carcinoma cells (A2780) with IC_50_ values in the low micromolar range. The antiproliferative effect of **Fe1** was even higher than cisplatin. Interestingly, **Fe1** showed low in vivo toxicity, and in vivo analyses of **Fe1** and **Fe2** compounds using colorectal HCT116 zebrafish xenograft showed that both reduce the proliferation of human HCT116 colorectal cancer cells in vivo.

## 1. Introduction

Approximately 50% of chemotherapeutic regimens for the treatment of cancer are based on *cis*-platinum and its derivatives [1]. Although these compounds have achieved significant clinical benefits for some solid tumours, their efficacy has been limited by toxic side effects and the development of drug resistance [2,3,4]. However, their proven antitumor activity is driving the search for new metal complexes as potential anticancer drugs [5,6,7,8]. Among these new complexes, *N*-heterocyclic carbene (NHC) metal complexes have recently gained considerable attention due to their inherent properties; they are readily available, and their physicochemical properties are easily fine-tuned by modification of the wingtip *N*-substituents, allowing the development of highly stable metal complexes [9]. In addition, the facile access to water-soluble NHC ligands represents an attractive feature for the development of new bioactive metal NHC compounds [10,11]. Recently, the antiproliferative activity of NHC complexes of Ag, Au, Pt, Pd, Cu, Ir, Rh, and Ru have been reported showing promising pharmacological properties as new antitumor drugs [9,12,13]. Some of them exhibit cytotoxic effects comparable to cisplatin and are capable of inducing apoptosis. Interestingly, several Ag(I), Au(III), Pt(II), Pd(II), and Rh(I) NHC complexes have been shown to be effective in vivo [9,12,14]. These recent achievements demonstrate the great potential of metal–NHC complexes as antitumor agents.

On the other hand, iron complexes have recently attracted the interest of inorganic medicinal chemists for the development of anticancer agents [15]. The anticancer activity of iron compounds, such as Fe–photocytotoxic complexes [16,17], Fe–multinuclear complexes [18], ferrocenyl derivatives [19], salen compounds [20,21,22], iron cyclopentadienyl [23], and iron polypyridyl complexes [24], has been reported in recent years. Among them, ferrocene derivatives are the most studied; both ferroquine and ferrocifen have been intensively investigated as anticancer drugs [25]. It has been demonstrated that the ferrocenyl fragment in these species has a crucial role in their therapeutic effect [25]. As an extension of the excellent results obtained with metallocenes, half-sandwich iron(II) compounds have received recent attention [26,27]. In particular, diiron carbonyl cyclopentadienyl complexes bearing different co-ligands such as aminocarbyne/iminium [28] or vinyliminum ligands [29,30] represent a suitable class of organometallic anticancer compounds. They have shown ideal properties such as the presence of a nontoxic metal, appreciable water solubility and amphiphilicity, and stability in aqueous media [28,29,30]. Despite the intensive research on iron complexes as possible anticancer drugs, the medicinal potential of Fe–NHC complexes has remained almost unexplored [31]. However, it has been proved that NHC ligands can be very useful for the stabilisation of iron organometallic complexes [32,33,34].

**Figure 1:**
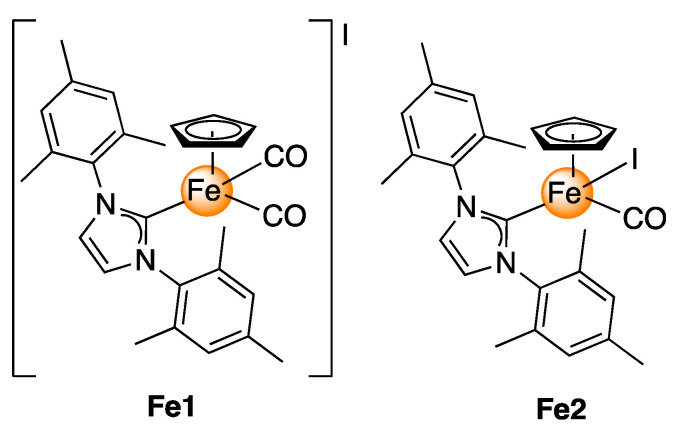
Molecular structure of the Fe–NHC complexes tested in this work.

Motivated by our interest in developing new effective anticancer metal-based drugs [35,36,37] and considering the recent aptitudes of NHC ligands in drug design, the known anticancer activity of iron compounds [25,26,27,28,29,30,31], and our large experience in Fe–NHC organometallic chemistry [38,39,40,41], we decided to study the anticancer activity of half-sandwich Fe–NHC compounds. As an NHC, we chose the bulky 1,3-bis-(2,4,6-trimethylphenyl)imidazol-2-ylidene (IMes) ligand. We rationalised that the presence of the sterically hindered IMes ligand would impart steric protection to the metal centre, thus enhancing the stability of the Fe complexes. Initially, we explored the antiproliferative activity of two Fe–NHC complexes, the cationic **Fe1** and the neutral **Fe2** complexes, shown in Figure 1, by assessing their in vitro activity against human cancer cell lines. In addition, the antiproliferative activity of **Fe1** and **Fe2** in human dermal fibroblasts and the in vivo toxicity and the xenograft in the zebrafish model were performed to evaluate the toxicological profile of these compounds. To the best of the authors’ knowledge, this work represents the first in vivo study of CpFe-NHC complexes as anticancer drugs.

## 2. Results and Discussion

### 2.1. Synthesis

The iron(II) complexes [Cp(IMes)Fe(CO)_2_]I (**Fe1**) and Cp(IMes)Fe(CO)I (**Fe2**) (IMes = 1,3-bis(2,4,6-trimethyl-phenyl)imidazol-2-ylidene) were synthesised following the procedures previously described in the literature [42,43].

### 2.2. Stability of the Complexes

**Figure 2:**
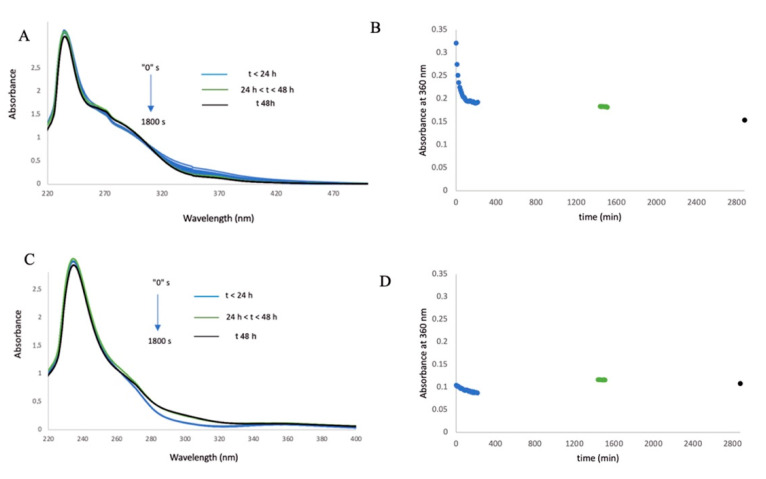
Time evolution of the UV–vis absorption spectrum of **Fe1** (**A**) and **Fe2** (**C**) in PBS buffer at pH 7 at room temperature under aerobic conditions. Variation of the absorbance, measured at 360 nm of **Fe1** (**B**) and **Fe2** (**D**). Blue colour indicates measurements made at t < 24 h, green colour indicates measurements at 24 h < t < 48 h, and black colour indicates measurements at t 48 h.

Complexes **Fe1** and **Fe2** are insoluble in buffer aqueous media. Solubility was fully achieved by adding DMSO to their buffer aqueous solutions, assuring that the final concentration of DMSO does not exceed 0.1% *v*/*v* (critical for further biological experiments). The stability of complexes **Fe1** and **Fe2** was evaluated over 48 h at room temperature by monitoring their UV–visible spectrum in PBS (pH 7.0) (Figure 2). As shown in Figure 2B, a rapid decrease of the intensity of the 360 nm band of complex **Fe1** occurs in the first 30 min, attaining a final absorbance value that is maintained at 24 h, slightly decreasing at 48 h. This behaviour suggests that **Fe1** is rapidly transformed into a new Fe species that is then stable, at least, until 24 h. In the case of **Fe2** (Figure 2D), a lower decrease of the 360 nm peak intensity occurs within the first 30 min (compared to **Fe1**), and a slight increase in the absorbance is observed at 24 h (that is maintained at 48 h). Based on our previous works with Ru carbonyl metal complexes [44], we speculate that release of CO ligand, 2 in the case of **Fe1** and 1 in the case of **Fe2**, may occur in the first 30 min, as the decrease profile is more pronounced for **Fe1** with two CO ligands (Figure 2B,D). We could also speculate that, in the case of **Fe1**, both CO ligands might be replaced by water molecules, maintaining the previous charge on this novel Fe species (cationic), while in **Fe2**, the release of the CO ligand and replacement by water molecule would maintain this new Fe species neutral.

### 2.3. Cytotoxic Activity

The in vitro cytotoxic potential of complexes **Fe1** and **Fe2** was evaluated through the CellTiter 96^®^Aqueous Non-Radioactive Cell Proliferation Assay using MTS, as described in Section 3.3.2 [26].

**Figure 3:**
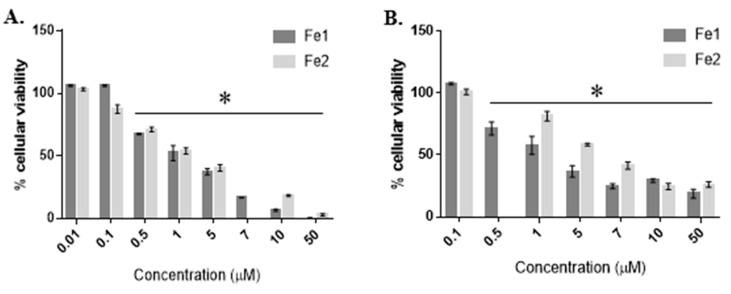
Cell viability (%) of A2780 (**A**) and HCT116 (**B**) tumour cell lines after 48 h of exposure to **Fe1** and **Fe2** complexes. Cell viability was determined using the MTS assay. Data expressed as mean ± SEM. * *p* < 0.05.

The antiproliferative activity of both complexes was evaluated through the exposure of the A2780 cell line to 0.01–50 µM of complexes **Fe1** or **Fe2** (Figure 3A) and of the HCT116 cell line to 0.1–50 µM of complexes **Fe1** or **Fe2** (Figure 3B) for 48 h. Figure 3 clearly shows a concentration-dependent reduction of cell viability in both tumour cell lines.

**Figure 4:**
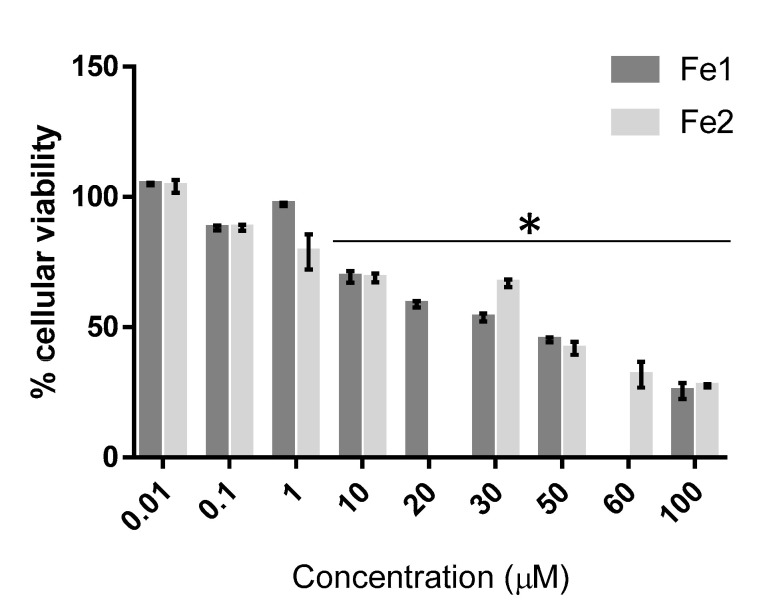
Cell viability (%) after 48 h of exposure of fibroblasts to the **Fe1** and **Fe2** complexes. Cell viability was determined using the MTS assay. Data expressed as mean ± SEM. * *p* < 0.05.

The antiproliferative effect of complexes **Fe1** and **Fe2** was also tested on a nontumor cell line, fibroblasts, to evaluate their antiproliferative activity in healthy cells. For that, a range of concentrations from 0.01 to 100 µM of both complexes was used (Figure 4). As previously observed for the tumour cell lines, a concentration-dependent reduction of cell viability is observed (Figure 3 and Figure 4).

The IC_50_ is a parameter that evaluates the effect of the tested complexes and indicates the complex concentration necessary to inhibit 50% of cellular proliferation [45,46]. Moreover, to evaluate the selectivity of a complex towards tumour cells (compared to normal cells), we assessed the selectivity index (SI), which is based on the ratio of the IC_50_ of a complex in fibroblasts to the IC_50_ of the complex in a tumour cell line. Higher SI indicates that the complex is more selective for a particular tumour cell line [46]. The IC_50_ and SI values for both complexes in the studied cell lines are shown in Table 1.

As observed in Table 1, complex **Fe1** shows the lowest relative IC_50_ in the HCT116 cell line (0.87 µM), demonstrating its high antiproliferative effect in this type of tumour cell line. Moreover, complex **Fe1** is also very active in the A2780 tumour cell line with a relatively low IC_50_ value of 1.34. Interestingly, complex **Fe1** has a high SI (27.1) for HCT116, compared to fibroblasts (with an IC_50_ of 23.61 µM)), demonstrating its selectivity towards colorectal cancer cells, compared to normal cells. On the other hand, the IC_50_ values for complex **Fe2** despite being higher, compared to complex **Fe1**, are also in the low micromolar range (IC_50_ of 2.91 µM in the A2780 cell line and 3.26 µM in HCT16 cell line). Complex **Fe2** presents a grade of selectivity considerably good in both cancer cell lines, with SI of 14.4 and 12.9 for A2780 and HCT116, respectively. Despite the change in absorbance spectra over time for complexes **Fe1** and **Fe2** in aqueous solution (PBS—Figure 2B,D), the cytotoxic effects attained at 48 h for both Fe species are reproducible for the triplicate biological assays (no major error bars and standard deviations are observed in Figure 3 and Table 1), and the Fe species exerting the cytotoxic effect might be distinct between **Fe1** and **Fe2** as the relative IC_50_ values in Table 1 and different response with a concentration in Figure 3 are different for complex **Fe1** and **Fe2** derived species. Moreover, we can speculate that **Fe1** might more easily release the two CO ligands forming a different Fe species, as a result of both ligand exchange (e.g., by water molecules) that could maintain the cationic charge and be more easily internalised justifying its higher cytotoxicity.

**Figure 5:**
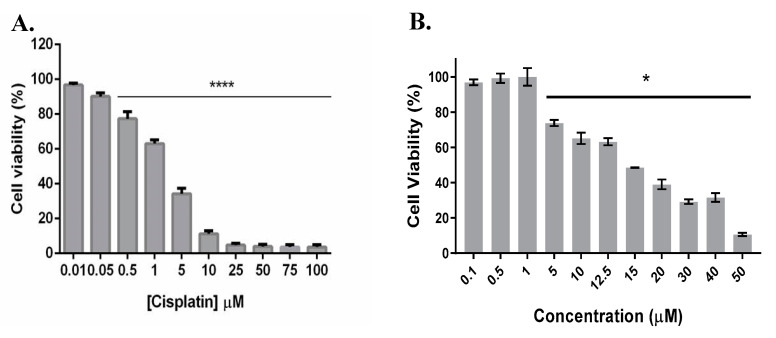
Cytotoxicity of cisplatin in A2780 (**A**) and HCT116 (**B**) cell lines after 48 h of incubation (1.9 ± 0.2 µM and 15.6 ± 5.3 µM for A2780 and HCT116 cell lines, respectively). Cell viability was determined using the MTS assay. Data normalised against the control (0.1% (*v*/*v*) DMSO) and expressed as the mean ± SEM of three independent assays. * *p* < 0.05; **** *p* < 0.005.

The therapeutic potential of complexes **Fe1** and **Fe2** was compared to the common chemotherapeutic agent already approved for clinical practice, cisplatin. The antiproliferative effect of cisplatin is shown in Figure 5. The IC_50_ results for cisplatin in A2780 and HCT116 cell lines are shown in Table 1. The antiproliferative activity in A2780 cells increases in the order **Fe1** > cisplatin > **Fe2**. On the other hand, in the case of the HCT116 cell line, cisplatin shows the highest IC_50_ value (15.6 µM), which means that cisplatin is the least cytotoxic compound for that cell line. Complexes **Fe1** and **Fe2** show high cytotoxicity in the HCT116 cell line (IC_50_ of 0.87 µM and 3.26 µM, respectively), which leads to a high therapeutical potential (in vitro) since at those concentrations, the cellular viability of fibroblasts (nontumor cell line) is not compromised.

### 2.4. In Vivo Toxicity Study: Fish Embryo Acute Toxicology Test (FET)

Zebrafish is currently considered an excellent model organism, compared to more complex organisms, due to its high fecundity, small size, and optical transparency [47]. In addition, zebrafish shares more than 70% of the genes with humans indeed [48]. To better comprehend the mechanisms of toxicity of chemical compounds, and to discover possible drugs for the treatment of human diseases such as cancer, zebrafish toxicity studies are adequate [49]. The toxicity study of **Fe1** and **Fe2** in vivo will provide reliable evidence for the safety of using these compounds as anticancer agents.

**Figure 6:**
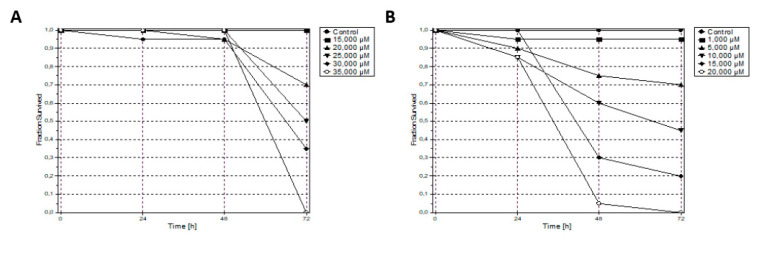
Accumulative mortality over (72 h) for complex **Fe1** (**A**) and complex **Fe2** (**B**) at the different stages evaluated (in hpf).

**Figure 7:**
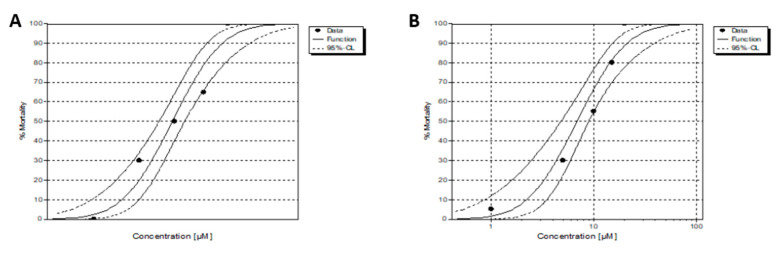
Mortality–response curve for **Fe1** (**A**) and **Fe2** (**B**).

As shown in Figure 6, fish survival data demonstrate that mortality is entirely dependent on Fe compound concentration. In addition, the mortality distribution obtained for each compound allowed the accurate estimation of the LC_50_ (Figure 7). Both **Fe1** and **Fe2** compounds showed moderate toxicity in vivo, being **Fe1** the least toxic (see Table 2). These results demonstrate the potential of cyclopentadienyl iron(II) NHC complexes as antiproliferative agents for ovarian and colon cancer therapy. For this reason, **Fe1** and **Fe2** compounds were analysed in vivo using colorectal HCT116 zebrafish xenograft.

### 2.5. Zebrafish Xenograft Assays

The zebrafish (*Danio rerio*) is a model organism that has become a very powerful tool for the study of human cancer over the past years. It is frequently used to test drugs for their cytotoxic or antimetastatic properties and study cell invasiveness. Zebrafish have much to offer, as they are cheap, easy to work with, and the embryonic model is relatively easy to use in high-throughput assays [50]. Taking this into account, it is, therefore, a useful model for human cancer cell xenotransplantation and toxicity studies of different chemotherapeutic compounds in vivo [51].

Interestingly, it was observed that **Fe1** and **Fe2** compounds reduce the proliferation of human HCT116 colorectal cancer cells in vivo. HCT116 human colorectal cancer cell line was incubated in standard conditions in a humidified incubator at 37 °C and 5% CO_2_ until a confluence of 70–80% was reached. Afterwards, the cells were concentrated and labelled with DiI and injected in the circulation (Duct of Cuvier) of 48 hpf zebrafish embryos in order to assay the proliferation in vivo of the cells exposed to **Fe1** and **Fe2**. **Fe1** and **Fe2** (5 μM and 0.25 μM, respectively) were dissolved in the water at 1-day post injection (dpi) to allow the recovery of the embryos from the injection in the first 24 h and maintained until the end of the experiment at 3 dpi. Imaging of the embryos was performed at 1 dpi and 3 dpi to quantify the fluorescence and area of the cells in the caudal hematopoietic tissue (CHT) in the tail of the embryos, where cells invade and grow, and measure their proliferation over time to assess the inhibitory effect of the compounds assayed over the cells.

**Figure 8:**
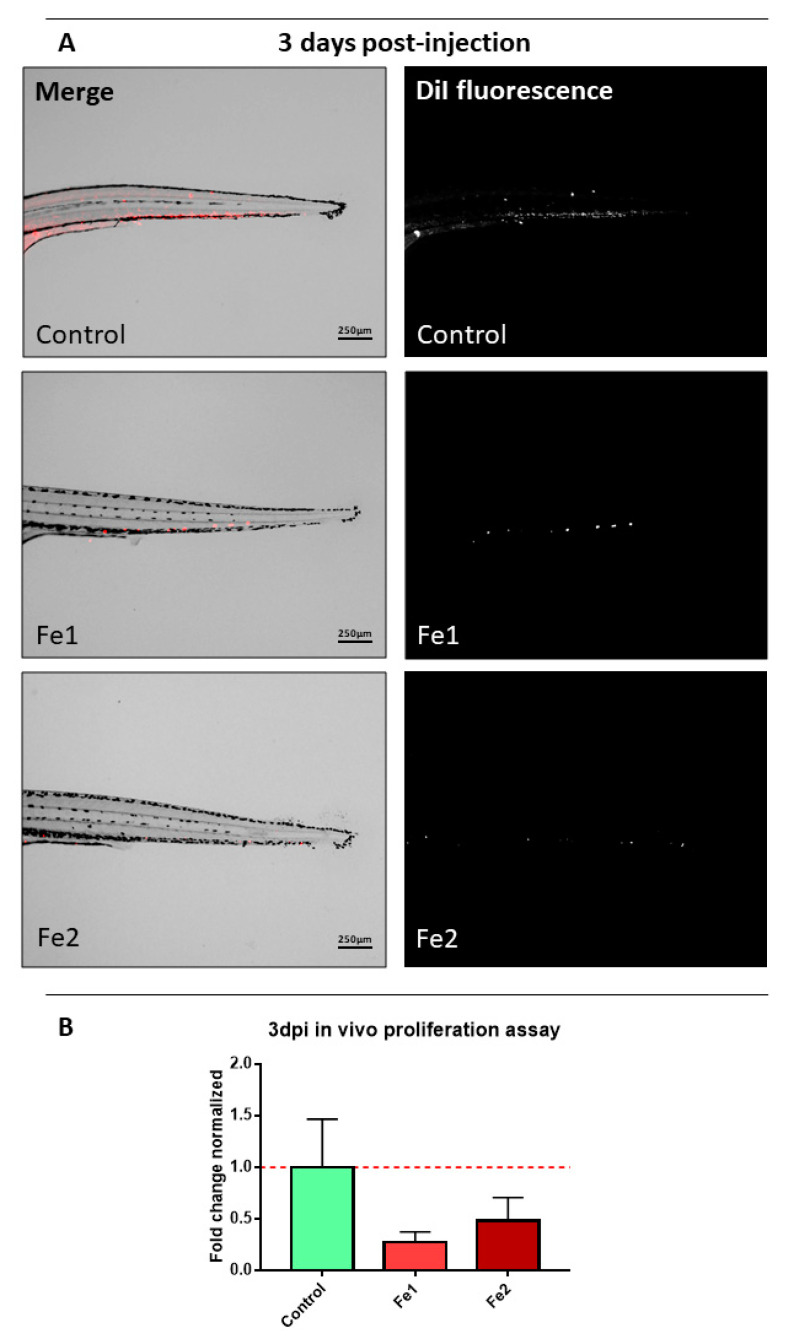
In vivo effectivity assays of **Fe1** and **Fe2** against human HCT116 colorectal cancer cell line: (**A**) Representative images of the caudal hematopoietic tissue (CHT) in the tail region of the zebrafish embryos where cells metastasize and proliferate at 3 dpi. Main images are a superposition of a fluorescence image and a bright field image of the same embryo. Fluorescence images show only the labelled cells of the main image. Scale = 250 µm; (**B**) fold change comparing 3 dpi against 1 dpi and normalised to the control condition at 3 dpi. The red line state the cell maintenance, above the bar, proliferation occurs, and below the bar, the cells decrease, compared to the control condition (n = 6 embryos/condition).

The obtained results indicate that **Fe1** and **Fe2** compounds that are dissolved in the water of the zebrafish embryos during incubation have great potential as anticancer agents due to their capacity of decreasing the proliferation of the cells at 3 dpi, compared with the control condition (Figure 8). The results were calculated based on a comparison between 3 dpi and 1 dpi time points and normalised at 3 dpi against control conditions due to the nature of the lipophilic dye in the proliferation of the cells.

## 3. Materials and Methods

### 3.1. Synthesis

Compounds **Fe1** and **Fe2** were prepared according to previously described procedures [42,43].

### 3.2. Stability of the Complexes

Complexes **Fe1** and **Fe2** solutions were prepared in 10 mM phosphate-buffered solution (PBS) containing 0.15 M NaCl (pH 7). All spectra were recorded for 48 h on a recorded with a Varian-Cary 100 Bio spectrophotometer (Agilent, Santa Clara, CA, USA), using a quartz cuvette with 1 cm path length in a wavelength range from 220 to 500 nm.

### 3.3. Cytotoxic Activity

#### 3.3.1. Cell Culture

Human colorectal carcinoma (HCT116) and normal dermal fibroblasts cell lines were grown in Dulbecco’s modified Eagle’s medium (DMEM) (Invitrogen Corp., Grand Island, NY, USA) supplemented with 10% (*v*/*v*) foetal bovine serum and 1% (*v*/*v*) antibiotic/antimycotic solution (Invitrogen Corp.). Human ovarian carcinoma (A2780) cell line was cultivated using the Roswell Park Memorial Institute (RPMI) medium supplemented as DMEM. The supplemented medium is named complete medium. Cells were grown in an incubator with a humidified atmosphere at 5% (*v*/*v*) CO_2_ and 37 °C. All cell lines were purchase from ATCC (ATCC^®^, American Type Culture Collection, Washington, DC, USA).

#### 3.3.2. Cell Viability

The evaluation of the cytotoxic potential of the complexes **Fe1** and **Fe2** was obtained through the measurement in vitro of the antiproliferative activity of these complexes. Cells were plated in 96-well plates at 7.5 × 10^4^ cells/mL and incubated at 37 °C with 5% (*v*/*v*) CO_2_. After 24 h, the culture medium was replaced with a fresh medium containing 0.01–100 µM of complexes **Fe1** or **Fe2** and incubated for 48 h, as described previously. DMSO 0.1% (*v*/*v*) was used as the vehicle control for all cell lines. The fibroblasts cell line was used as a control of cytotoxicity of the complexes for healthy cells. Cisplatin was used as a positive control (a common chemotherapeutic agent). Cell viability was evaluated using CellTiter 96^®^Aqueous Non-Radioactive Cell Proliferation Assay (Promega, Madison, WI, USA) using 3-(4,5-dimethylthiazol-2-yl)-5-(3-carboxymethoxyphenyl)-2-(4-sulfophenyl)-2H-tetrazolium, inner salt (MTS), as described previously [27,28]. In metabolically active cells, enzymes present in the mitochondria catalyse a reaction in which NADPH/ NADH is produced. These enzymes can reduce the MTS reagent into a brownish-coloured product called formazan, which can be quantified by measuring the absorbance at 490 nm. The quantity of product produced is directly proportional to the number of viable cells in culture. The amount of formazan product formed was measured with a Bio-Rad Microplate Reader Model 680 (Bio-Rad, Hercules, CA, USA). Half maximal inhibitory concentration (IC_50_) was calculated using GraphPad Prism 6 (GraphPad Software, La Jolla, CA, USA). Selectivity index (SI) was calculated for both tumour cell lines by dividing the IC_50_ of fibroblasts with the IC_50_ of HCT116 or A2780 for complexes **Fe1** and **Fe2**. This selectivity index represents the selectivity of a complex towards tumour cells versus normal cells, and the higher the value is, the more active is the complex towards tumour cells, compared to normal cells.

### 3.4. In Vivo Toxicity Study: Fish Embryo Acute Toxicology Test (FET)

Zebrafish embryos and larvae below 120 hpf are not protected animal stages according to EU Directive 2010/63/EU and, therefore, do not fall within the regulatory frameworks related to animal experimentation [49]. However, the experiments considered the principles for human–animal research [52] and recommendations of the Spanish regulations (RD 53/2013).

The wild-type zebrafish were housed in a water recirculation system under controlled physicochemical conditions of temperature (26 ± 2 °C), pH (7–7.5), and conductivity (400–600 µS/cm). The fertilised eggs were collected after natural spawning and housed in Petri dishes until their use. Next, eggs were exposed to **Fe1** and **Fe2** and dissolved in water containing 1% DMSO. Then, toxicological analyses were carried out based on the approved standard OECD TG 236—fish embryo toxicity test (FET) [53], with modifications. For the study, four replicates of 12 embryos were distributed for each of the 5 concentrations tested for compounds; for **Fe1,** the concentrations used were 15, 20, 25, 30, 35 µM), and for **Fe2** were 1, 5, 10, 15, 20 µM. The performed zebrafish toxicity experiments were approved by the animal care and use committee of the University of Santiago de Compostela and the standard protocols of Spain (CEEA-LU-003 and Directive 2012-63-EU). The toxicity results data were analysed by probit analysis using ToxRat Software (ToxRat Solutions. 2003. ToxRat. Software for statistical analysis of bioassays. Alsdorf, Germany).

### 3.5. Zebrafish Handling and Care

The wild-type zebrafish adults were kept in 30 L aquariums in a water recirculation system with controlled light and temperature: light–dark photoperiod of 14/10 h, respectively, temperature 26 ± 2 °C, pH 7–7.5, and a conductivity of 400–600 µS/cm [54]. Adult zebrafish (*Danio rerio*, wild type) were crossed to obtain zebrafish embryos. The embryos were obtained by means of massive natural spawning, they were collected and placed in Petri dishes for 48 h until they were injected.

All the procedures used in the experiments, fish care, and treatment were performed in agreement with the animal care and use committee of the University of Santiago de Compostela and the standard protocols of Spain (Directive 2012-63-DaUE). At the end of the experiments, zebrafish embryos were euthanised by tricaine overdose.

#### 3.5.1. Cell Culture

The human colorectal carcinoma cell line (HCT116) was maintained in a humidified incubator at 37 °C and 5% CO_2_. The colon cancer cells were cultured in DMEM supplemented with 10% FBS and penicillin–streptomycin (PS) (100 U/mL each). Cells were collected at approximately 80% confluence.

#### 3.5.2. Zebrafish Embryo Xenograft Assays

Zebrafish embryos were collected and incubated at 28 °C during the first 48 h post fertilisation (hpf). At 48 hpf the embryos were anaesthetised with 0.003% tricaine (Sigma). HCT116 human colorectal carcinoma cells were trypsinised and one million cells were harvested and labelled with DiI lipophilic dye and concentrated in 10 μL of phosphate-buffered saline (PBS) containing 2% polyvinyl–pyrrolidone 40 (PVP40) to avoid cell aggregation.

Borosilicate needles (1 mm O.D. × 0.75 mm I.D.; World Precision Instruments, Sarasota, FL, USA) were used to perform the xenograft assays in the zebrafish embryos. Between 100 and 150 cells were injected into the circulation of each fish (duct of Cuvier) using a microinjector (IM-31 Electric Microinjector, Narishige, Tokio, Japan) with an output pressure of 34 kPA and 30 ms of injection time per injection. Afterwards, injected embryos were incubated at a temperature of 34 °C for 3 days post injection (dpi) in 20 mL Petri dishes for each condition with SDTW (salt dechlorinate tap water).

At 1 dpi, the embryos were imaged, and the compound (**Fe1** and **Fe2**) was added to the water. The concentrations used were 5 μM (**Fe1**) and 0.25 μM (**Fe2**), based on previous experiments in order not to harm the embryos during the experiment. Embryos were incubated for two more days (until 3 dpi) and imaged for the last time for image analysis by comparison of the two time points. Imaging of the embryos was performed using a fluorescence stereomicroscope (AZ-100, Nikon, Tokyo, Japan).

#### 3.5.3. Zebrafish Embryo Image Analysis

The analysis of the images taken at two different times to track the progression of the injected cells was performed with Quantifish Software 2.1.1 (University College, London, UK) [55]. This software analyses each image measuring the fluorescence of each image above a manually established threshold, yielding the integrated intensity of each image as an output. This measure is the result of multiplying the number of positive pixels by the medium intensity of the fluorescence in each image. The integrated intensity was the value further processed to obtain a proliferation ratio and compare the treatment (**Fe1** and **Fe2**) against the control HCT116 cells without compound added to the embryos (control).

## 4. Statistical Analysis

Graphpad Software (GraphPad Prism version 7.00 for Windows, GraphPad Software, La Jolla, CA, USA, www.graphpad.com) was used to perform the statistical analysis of the data performing a comparison using *t*-test with a confidence interval of 95%.

## 5. Conclusions

Both **Fe1** and **Fe2** show in vitro antiproliferative activity against HCT116 and A2780 cell lines, having **Fe1** the highest cytotoxic potential. Furthermore, the **Fe1** complex shows a high selectivity index for HCT116 and low toxicity in vivo, and it is the most active in vivo. Interestingly, both **Fe1** and **Fe2** complexes were active in vivo, reducing the proliferation of HCT116 cells. Thus, **Fe1** and **Fe2** have the potential as chemotherapeutic agents against colon cancer, laying the foundations for further biological studies such as in vivo mice xenograft studies to validate their antitumour capacity.

## Figures and Tables

**Table 1:** Relative IC_50_ of complexes **Fe1** and **Fe2** and of cisplatin on the A2780, HCT116, and fibroblasts cell lines. The selectivity index (SI) of the complexes is also presented. Data expressed as mean ± SEM.

Compound	Cell lines	IC_50_ (µM)	SI
**Fe1**	A2780	1.34 ± 0.25	17.7
	HCT116	0.87 ± 0.09	27.1
	Fibroblasts	23.61 ± 0.14	-
**Fe2**	A2780	2.91 ± 0.51	14.4
	HCT116	3.26 ± 0.10	12.9
	Fibroblasts	42.04 ± 0.75	-
Cisplatin	A2780	1.9 ± 0.2	-
	HCT116	15.6 ± 5.3	-

**Table 2:** Lethal concentration values (LC_50_ (µM)) of zebrafish embryos subjected to tested compounds from early blastula stage up to 72 hpf calculated by Logit and Probit analyses.

Compound	LC_50_ (µM)	C.L ^1^ (95%)	NOEC ^2^ (µM)	LOEC ^3^ (µM)
**Fe1**	24.624	22.653–26.622	15	20
**Fe2**	6.861	4.777–8.941	1	5

^1^ C.L Confidence limits; ^2^ NOEC (no observed effect concentration); ^3^ LOEC (lowest observed effect concentration).

## Data Availability

Not applicable.

## Sample Availability

Samples of complexes **Fe1** and **Fe2** are available from the authors.

## References

[1] Kelland L. (2007). The resurgence of platinum-based cancer chemotherapy. Nat. Rev..

[2] Wilson J.J., Lippard S.J. (2014). Synthetic Methods for the Preparation of Platinum Anticancer Complexes. Chem. Rev..

[3] Dasari S., Tchounwou P.B. (2014). Cisplatin in cancer therapy: Molecular mechanisms of action. Eur. J. Pharmacol..

[4] Ghosh S. (2019). Cisplatin: The first metal based anticancer drug. Bioorg. Chem..

[5] Pedrosa P., Carvalho A., Baptista P.V., Fernandes A.R. (2018). Inorganic Coordination Chemistry: Where We Stand in Cancer Treatment?. Basic Concepts Viewed from Frontier in Inorganic Coordination Chemistry.

[6] Jia P., Ouyang R., Cao P., Tong X., Zhou X., Lei T., Zhao Y., Guo N., Chang H., Miao Y. (2017). Review: Recent Advances and Future Development of Metal Complexes as Anticancer Agents. J. Coord. Chem..

[7] Anthony E.J., Bolitho E.M., Bridgewater H.E., Carter O.W.L., Donnelly J.M., Imberti C., Lant E.C., Lermyte F., Needham R.J., Palau M. (2020). Metallodrugs are unique: Opportunities and challenges of discovery and development. Chem. Sci..

[8] Boros E., Dyson P.J., Gasser G. (2020). Classification of metal-based drugs according to their mechanisms of action. Chem.

[9] Liu W., Gust R. (2013). Metal N-Heterocyclic Carbene Complexes as Potential Antitumor Metallodrugs. Chem. Soc. Rev..

[10] Schaper L.-A., Hock S.J., Herrmann W.A., Kühn F.E. (2013). Synthesis and application of water-soluble NHC transition-metal complexes. Angew. Chem. Int. Ed..

[11] Gautier A., Cisnetti F. (2012). Advances in Metal-Carbene Complexes as Potent Anti-Cancer Agents. Metallomics.

[12] Tong K.-C., Hu D., Wan P.-K., Lok C.-N., Che C.-M. (2020). Anti-cancer gold, platinum and iridium compounds with porphyrin and/or N-heterocyclic carbene ligand(s). Medicinal Chemistry.

[13] A Patil S., P Hoagland A., A Patil S., Bugarin A. (2020). N-Heterocyclic Carbene-Metal Complexes as Bio-Organometallic Antimicrobial and Anticancer Drugs, an Update (2015–2020). Future Med. Chem..

[14] Guarra F., Pratesi A., Gabbiani C., Biver T. (2021). A focus on the biological targets for coinage metal-NHCs as potential anticancer complexes. J. Inorg. Biochem..

[15] Bouché M., Hognon C., Grandemange S., Monari A., Gros P.C. (2020). Recent Advances in Iron-Complexes as Drug Candidates for Cancer Therapy: Reactivity, Mechanism of Action and Metabolites. Dalton Trans..

[16] Basu U., Pant I., Khan I., Hussain A., Kondaiah P., Chakravarty A.R. (2014). Iron(III) Catecholates for Cellular Imaging and Photocytotoxicity in Red Light. Chem. Asian J..

[17] Saha S., Majumdar R., Roy M., Dighe R.R., Chakravarty A.R. (2009). An Iron Complex of Dipyridophenazine as a Potent Photocytotoxic Agent in Visible Light. Inorg. Chem..

[18] Sanina N.A., Kozub G.I., Zhukova O.S., Emelyanova N.S., Kondrateva T.A., Korchagin D.V., Shilov G.V., Ovanesyan N.S., Aldoshin S.M. (2013). Synthesis, Structure, NO Donor Activity of Iron-Sulfur Nitrosyl Complex with 2-Aminophenol-2-Yl and Its Antiproliferative Activity against Human Cancer Cells. J. Coord. Chem..

[19] Jaouen G., Vessières A., Top S. (2015). Ferrocifen type anti-cancer drugs. Chem. Soc. Rev..

[20] Lee S.-Y., Hille A., Kitanovic I., Jesse P., Henze G., Wölfl S., Gust R., Prokop A. (2011). [Fe(III)(salophene)Cl], a potent iron salophene complex overcomes multiple drug resistance in lymphoma and leukemia cells. Leuk. Res..

[21] Vančo J., Šindelář Z., Dvořák Z., Trávníček Z. (2015). Iron-salophen complexes involving azole-derived ligands: A new group of compounds with high-level and broad-spectrum in vitro antitumor activity. J. Inorg. Biochem..

[22] Ghanbari Z., Housaindokht M.R., Izadyar M., Bozorgmehr M.R., Eshtiagh-Hosseini H., Bahrami A.R., Matin M.M., Khoshkholgh M.J. (2014). Structure-activity relationship for Fe(III)-salen-like complexes as potent anticancer agents. Sci. World J..

[23] Florindo P.R., Pereira D.M., Borralho P.M., Rodrigues C.M.P., Piedade M.F.M., Fernandes A.C. (2015). Cyclopentadi-enyl-ruthenium(II) and iron(II) organometallic compounds with carbohydrate derivative ligands as good col-orectal anticancer agentes. J. Med. Chem..

[24] Lin H., Wang Y., Lai H., Li X., Chen T. (2018). Iron(II)-Polypyridyl complexes inhibit the growth of glioblastoma tumor and enhance TRAIL-induced cell apoptosis. Chem. Asian J..

[25] Patra M., Gasser G. (2017). The medicinal chemistry of ferrocene and its derivatives. Nat. Rev. Chem..

[26] Pilon A., Brás A.R., Côrte-Real L., Avecilla F., Costa P.J., Preto A., Garcia M.H., Valente A. (2020). A new family of iron(II)-cyclopentadienyl compounds shows strong activity against colorectal and triple negative breast cancer cells. Molecules.

[27] Pilon A., Gírio P., Nogueira G., Avecilla F., Adams H., Lorenzo J., Garcia M.H., Valente A. (2017). New iron cyclopentadienyl complexes bearing different phosphane co-ligands: Structural factors vs. cytotoxicity. J. Organomet. Chem..

[28] Biancalana L., De Franco M., Ciancaleoni G., Zacchini S., Pampaloni G., Gandin V., Marchetti F. (2021). Easily available, amphiphilic diiron cyclopentadienyl complexes exhibit in vitro anticancer activity in 2D and 3D human cancer cells through redox modulation triggered by CO release. Chem. Eur. J..

[29] Rocco D., Batchelor L.K., Agonigi G., Braccini S., Chiellini F., Schoch S., Biver T., Funaioli T., Zacchini S., Biancalana L. (2019). Anticancer potential of diiron vinyliminium complexes. Chem. Eur. J..

[30] Braccini S., Rizzi G., Biancalana L., Pratesi A., Zacchini S., Pampaloni G., Chiellini F., Marchetti F. (2021). Anticancer diiron vinyliminium complexes: A structure-activity relationship study. Pharmaceutics.

[31] Cingolani A., Zanotti V., Zacchini S., Massi M., Simpson P.V., Maheshkumar Desai N., Casari I., Falasca M., Rigamonti L., Mazzoni R. (2019). Synthesis, reactivity and preliminary biological activity of iron(0) complexes with cyclopentadienone and amino-appended N-heterocyclic carbene ligands. Appl. Organomet. Chem..

[32] Riener K., Haslinger S., Raba A., Högerl M.P., Cokoja M., Herrmann W.A., Kühn F.E. (2014). Chemistry of iron N-heterocyclic carbene complexes: Syntheses, structures, reactivities, and catalytic applications. Chem. Rev..

[33] Johnson C., Albrecht M. (2017). Piano-stool N-heterocyclic carbene iron complexes: Synthesis, reactivity and catalytic applications. Coord. Chem. Rev..

[34] Selvakumar J., Simpson S.M., Zurek E., Arumugam K. (2021). An electrochemically controlled release of NHCs using iron bis(dithiolene) N-heterocyclic carbene complexes. Inorg. Chem. Front..

[35] Lenis-Rojas O.A., Robalo M.P., Tomaz A.I., Carvalho A., Fernandes A.R., Marques F., Folgueira M., Yáñez J., Vázquez-García D., López Torres M. (2018). RuII(p-cymene) compounds as effective and selective anticancer candidates with no toxicity in vivo. Inorg. Chem..

[36] Lenis-Rojas O.A., Roma-Rodrigues C., Fernandes A.R., Marques F., Pérez-Fernández D., Guerra-Varela J., Sánchez L., Vázquez-García D., López-Torres M., Fernández A. (2017). Dinuclear RuII(bipy)2 derivatives: Structural, biological, and in vivo zebrafish toxicity evaluation. Inorg. Chem..

[37] Lenis-Rojas O.A., Fernandes A.R., Roma-Rodrigues C., Baptista P.V., Marques F., Pérez-Fernández D., Guerra-Varela J., Sánchez L., Vázquez-García D., Torres M.L. (2016). Heteroleptic mononuclear compounds of ruthenium(ii): Synthesis, structural analyses, in vitro antitumor activity and in vivo toxicity on zebrafish embryos. Dalton Trans..

[38] Warratz S., Postigo L., Royo B. (2013). Direct synthesis of iron(0) N-heterocyclic carbene complexes by using Fe_3_(CO)_12_ and their application in reduction of carbonyl groups. Organometallics.

[39] da Costa A.P., Mata J.A., Royo B., Peris E. (2010). Preparation of cp-functionalized N-heterocyclic carbene complexes of ruthenium. Resolution of chiral complexes and catalytic studies. Organometallics.

[40] Cardoso J.M.S., Fernandes A., Cardoso B.D., Carvalho M.D., Ferreira L.P., Calhorda M.J., Royo B. (2014). Cationic half-sandwich iron(II) and iron(III) complexes with N-heterocyclic carbene ligands. Organometallics.

[41] Lopes R., Raya-Barón Á., Robalo M.P., Vinagreiro C., Barroso S., Romão M.J., Fernández I., Pereira M.M., Royo B. (2021). Donor functionalized iron(II) N-heterocyclic carbene complexes in transfer hydrogenation reactions. Eur. J. Inorg. Chem..

[42] Jiang F., Bézier D., Sortais J.-B., Darcel C. (2011). N-heterocyclic carbene piano-stool iron complexes as efficient catalysts for hydrosilylation of carbonyl derivatives. Adv. Synth. Catal..

[43] Buchgraber P., Toupet L., Guerchais V. (2003). Syntheses, properties, and X-ray crystal structures of piano-stool iron complexes bearing an N-heterocyclic carbene ligand. Organometallics.

[44] Fernandes A.R., Mendonça-Martins I., Santos M.F.A., Raposo L.R., Mendes R., Marques J., Romão C.C., Romão M.J., Santos-Silva T., Baptista P.V. (2020). Improving the anti-inflammatory response via gold nanoparticle vectorization of CO-releasing molecules. ACS Biomater. Sci. Eng..

[45] Ma Z., Zhang B., Guedes da Silva M.F.C., Silva J., Mendo A.S., Baptista P.V., Fernandes A.R., Pombeiro A.J.L. (2016). Synthesis, characterization, thermal properties and antiproliferative potential of copper(II) 4′-phenyl-terpyridine compounds. Dalton Trans..

[46] Czerwińska K., Machura B., Kula S., Krompiec S., Erfurt K., Roma-Rodrigues C., Fernandes A.R., Shul’pina L.S., Ikonnikov N.S., Shul’pin G.B. (2017). Copper(ii) complexes of functionalized 2,2′:6′,2″-terpyridines and 2,6-di(thiazol-2-yl)pyridine: Structure, spectroscopy, cytotoxicity and catalytic activity. Dalton Trans..

[47] Truong L., Harper S.L., Tanguay R.L. (2011). Evaluation of embryotoxicity using the zebrafish model. Methods Mol. Biol..

[48] Howe K., Clark M.D., Torroja C.F., Torrance J., Berthelot C., Muffato M., Collins J.E., Humphray S., McLaren K., Matthews L. (2013). The zebrafish reference genome sequence and its relationship to the human genome. Nature.

[49] (2010). DIRECTIVE 2010/63/EU OF THE EUROPEAN PARLIAMENT AND OF THE COUNCIL of 22 September 2010 on the protection of animals used for scientific purposes. https://eur-lex.europa.eu/LexUriServ/LexUriServ.do?uri=OJ:L:2010:276:0033:0079:en:PDF.

[50] Wyatt R.A., Trieu N.P.V., Crawford B.D. (2017). Zebrafish Xenograft: An Evolutionary Experiment in Tumour Biology. Genes.

[51] Cabezas-Sainz P., Guerra-Varela J., Carreira M.J., Mariscal J., Roel M., Rubiolo J.A., Sciara A.A., Abal M., Botana L.M., López R. (2018). Improving zebrafish embryo xenotransplantation conditions by increasing incubation temperature and establishing a proliferation index with ZFtool. BMC Cancer.

[52] Russell W.M.S., Burch R.L. (1959). The Principles of Humane Experimental Technique.

[53] (2009). OECD Guidelines for the Testing of Chemicals, Section 2: Effects on Biotic Systems.

[54] Westerfield M. (2000). The Zebrafish Book. A Guide for the Laboratory Use of Zebrafish (*Danio rerio*).

[55] Stirling D.R., Suleyman O., Gil E., Elks P.M., Torraca V., Noursadeghi M., Tomlinson G.S. (2020). Analysis tools to quantify dissemination of pathology in zebrafish larvae. Sci Rep..

